# Operational research leading to state-level policy change in tuberculosis undernutrition in Chhattisgarh, India

**DOI:** 10.5588/pha.26.0016

**Published:** 2026-08-24

**Authors:** C. Malik, V. Gupta, K. Shringarpure, H.A. Gupte, V.R. Keshri, M. Damani, N.N. Shah, K. Sonwani, P. Nanda, A. Sharma, S.K. Mattoo, H.D. Shewade

**Affiliations:** 1 Sangwari-People’s Association for Equity and Health, Surguja, Chhattisgarh, India;; 2 State Health Resource Centre, Raipur, Chhattisgarh, India;; 3 Department of Community Medicine, Medical College Baroda, Vadodara, Gujarat, India;; 4 Narotam Sekhsaria Foundation, Mumbai, Maharashtra, India;; 5 Jindal School of Public Health and Human Development, O. P. Jindal Global University, Sonipat, India;; 6 Department of Health and Family Welfare, National Tuberculosis Elimination Programme (NTEP), National Health Mission, Raipur, Chhattisgarh, India;; 7 Directorate of Health Services, Department of Health, Raipur, India;; 8 Office of the Chief Medical and Health Officer (CMHO), Khairagarh-Chhuikhadan-Gandai, Department of Health and Family Welfare, Government of Chhattisgarh, Chhattisgarh, India;; 9 Central TB Division, Ministry of Health and Family Welfare, Government of India, New Delhi, India;; 10 Division of Health Systems Research, ICMR National Institute of Epidemiology (ICMR-NIE), Chennai, India.

**Keywords:** tuberculosis, nutrition, BMI, food basket, malnutrition, M-PHISOR

## Abstract

In India, undernutrition is a key driver of the tuberculosis (TB) epidemic. The high burden of severe and very severe undernutrition leads to higher death rates among those with TB. Despite documentation of weight and height, body mass index calculations to prioritise nutrition support for those with severe and very severe undernutrition are lacking in India’s National Tuberculosis Elimination Programme. This state-level operational research in Chhattisgarh led to a change in state-level policy in this regard. The learnings from this operational research–guided policy change have lessons for other states in India.

The tuberculosis (TB) epidemic in India is driven by high rates of undernutrition, accounting for one third of the TB incidence.^1^ The burden of severe and very severe undernutrition among people with TB is high and they are 4–5 times more likely to die.^2^ The 2017 national guidance document on nutritional support in TB recommends body mass index (BMI)-based reporting of the burden of severe (BMI 14–14.9 kg/m^2^) and very severe undernutrition (BMI < 14 kg/m^2^) among adults with TB. It also recommends prioritisation of support for persons with TB with severe/very severe undernutrition. The suggested nutritional supplementation is doubling of the monthly family ration and additional food basket for such households.^3^ However, these recommendations have not been operationalised in India’s National Tuberculosis Elimination Programme (NTEP). The NTEP encourages nutrition support through food baskets (by volunteers) known as *Ni-kshay Mitras*. It also provides direct benefit transfer (DBT) of INR 1000 every month for people with TB.^4^ However, the programme managers are unaware of the burden and severity of undernutrition among people with TB. This is not documented or reported, and therefore, there are no mechanisms to implement or prioritise these existing schemes.

Starting in July 2024, a state-level operational research (OR) in the high-TB-burden central Indian state of Chhattisgarh was undertaken to understand and address the burden of undernutrition among adults with TB under programme settings in the public health system.^5^ The results led to state-level policy and practice change in December 2025 by implementing undernutrition monitoring using BMI and prioritising nutrition support schemes for those with severe and very severe undernutrition to be reported by all districts. We share our experience from the conception of this OR to the issuance of a state-level guidance, including the challenges faced and measures to address key programmatic gaps.

## ASPECTS OF INTEREST

OR is a medium of policy/practice change in health programmes. ICMR National Institute of Epidemiology (ICMR-NIE), Chennai, India, issued a call for applications for a national Structured Operational Research Training Initiative (SORT IT) course on TB.^6^ Applicants submitted concept notes for OR on priority research questions of the NTEP. A SORT IT course is for 1 year, where a selected mentee (n = 12 per course) undertakes a mentored OR project. On successful completion of year 1 (submission of manuscript to a peer-reviewed scientific journal), the mentee is awarded a SORT IT certificate.^7^ The second year under the course was Mentorship for Public Health Implementation Strengthening and Operational Research (M-PHISOR). On successful completion of another mentored OR project in year 2, as a follow-up of year 1 (SORT IT), an M-PHISOR certificate was awarded (an ICMR-NIE innovation). From Chhattisgarh, one candidate (VG) proposed to assess the burden of undernutrition among adults with TB and coverage of nutrition-related schemes in the state as year 1 OR. For adults notified with TB in the public health system, data captured in NTEP’s web-based case management system, *Ni-kshay*, on weight, height, receipt of DBT, and food basket were extracted to derive BMI and classify undernutrition. Access to DBT and food basket within 1 and 2 months was studied overall, and among those with severe/very severe undernutrition.^5^

**FIGURE 1.**
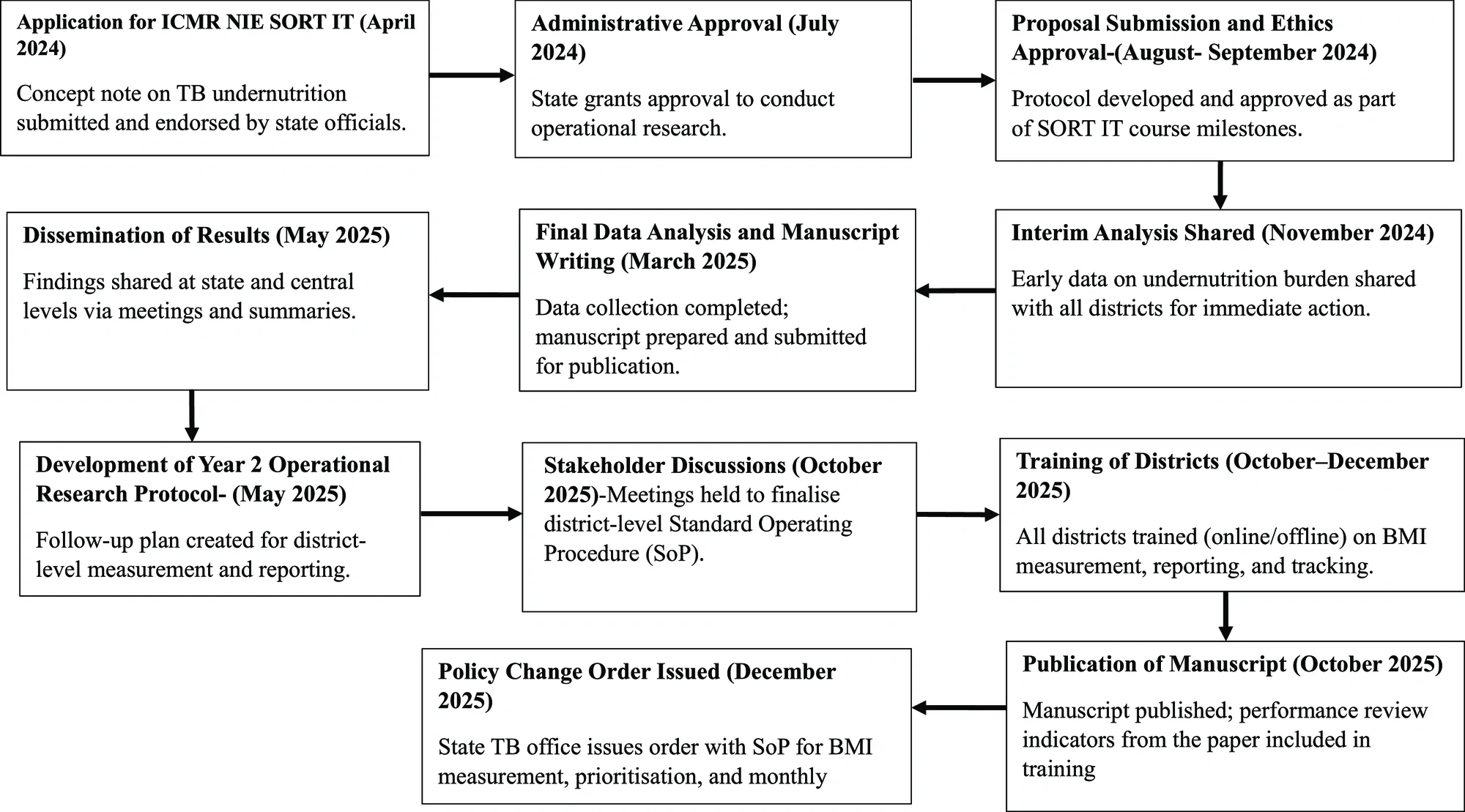
Key events and timeline of TB-undernutrition policy change using operational research in Chhattisgarh, India. TB = tuberculosis.

In year 1, OR revealed that 23% had severe and 14% had very severe undernutrition. DBT receipt within 2 months of notification was 29%, and food basket was received by only 21%. Inequity and delay in receipt among persons with various levels of undernutrition was observed.^5^ These results and recommendations to measure and monitor BMI and to identify and prioritise those with severe and very severe undernutrition for nutrition support schemes were shared with the state TB cell in a review meeting in May 2025. A reporting format to track TB-undernutrition was also shared. State-level deliberations led to a plan to implement recommendations of the year 1 OR in October 2025. The state TB office issued orders on 8 December 2025 for all districts to follow a standard operating procedure (SOP). This included directions on BMI calculation, BMI-based categorisation, prioritisation of DBT for those with severe and very severe undernutrition by updating bank account details in Ni-kshay within 24–48 h of notification, and ensuring DBT and food basket support within 15 days. The implementation of this SOP will be studied as the year 2 OR project. Key events have been summarised in Figure 1.

## DISCUSSION

**FIGURE 2.**
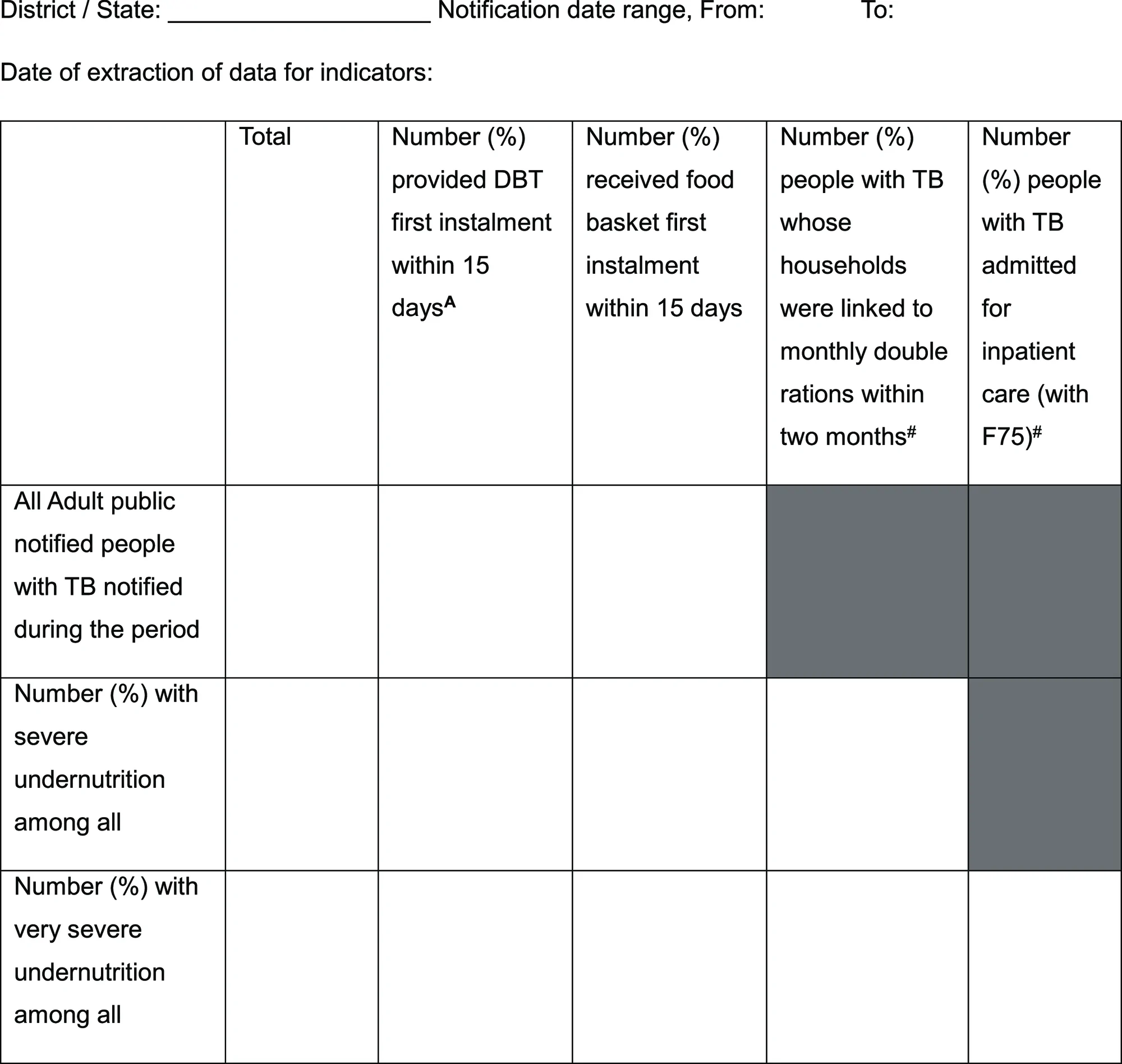
TB-undernutrition monitoring indicator included in state/district NTEP quarterly performance review, Chhattisgarh, India. ^A^All people with TB should be linked for DBT and food basket in line with NTEP guidance; ^B^Doubling of food rations and inpatient care for those with very severe undernutrition (BMI<14kg/m^2^) has been proposed to the state but is yet to be implemented. TB = tuberculosis; NTEP = National Tuberculosis Elimination Programme; BMI = body mass index; ICMR NIE = ICMR National Institute of Epidemiology; SORT IT = Structured Operational Research Training Initiative.

The state-level policy practice change was achieved within 18 months of the initiation of the OR in consultation with the state TB cell, ICMR-NIE, and other stakeholders. The following were the gaps identified and measures taken to bridge them (https://figshare.com/s/d14b9659e22e40f11e3f). 1) BMI reporting: The OR findings highlighted high burden of undernutrition among people with TB and the need for routine BMI measurement and reporting the burden. Thus, all districts were directed to ensure BMI assessment and reporting of the proportion of severe and very severe undernutrition. 2) DBT and food basket prioritisation: Inequity in the distribution of DBT and food basket support highlighted no mechanism that prioritised vulnerable individuals based on their BMI.^5^ The recommendation to prioritise people with severe and very severe undernutrition for nutrition support schemes was adopted. 3) Timeliness of nutrition support schemes: The findings revealed delays in DBT receipt. These delays and reasons have been documented previously.^8,9^ Based on this, clear timelines were defined to update bank account details and DBT and food basket provision for those with severe and very severe undernutrition. 4) Monitoring mechanism for TB-undernutrition: The OR recommended introduction of TB-undernutrition monitoring indicators.^5^ The state TB office adopted the reporting format (Figure 2) for districts to review and submit, as a programmatic monitoring mechanism previously lacking. 5) To conclude, the executive orders for adopting policy practice change will help in prioritisation and timely receipt of nutrition support schemes for those with severe and very severe undernutrition in the state and improve TB outcomes.

## CONCLUSION

The learnings from this experience of OR-guided policy change, where the state prioritised undernutrition in TB and addressed inequity in nutrition support schemes through programmatic action, have lessons for other states in India and for future work.
